# Assessing flexibility in meaning and context in non‐human communication

**DOI:** 10.1111/brv.70054

**Published:** 2025-07-23

**Authors:** Marlen Fröhlich, Juliette Aychet, Peter R. Clark, Catherine Crockford, Guillaume Dezecache, Nancy Rebout, Carel P. van Schaik, Kirsty E. Graham

**Affiliations:** ^1^ Paleoanthropology, Institute for Archaeological Sciences, Department of Geosciences University of Tübingen Tübingen 72076 Germany; ^2^ DFG Centre for Advanced Studies “Words, Bones, Genes, Tools” University of Tübingen Tübingen 72076 Germany; ^3^ Laboratoire Ethologie Cognition Développement (LECD, UR3456) Université Paris Nanterre Nanterre 92000 France; ^4^ Development and Behaviour Research Group, School of Psychology University of Lincoln Lincoln LN6 7TS UK; ^5^ CNRS Institute for Cognitive Sciences Marc Jeannerod Bron 69500 France; ^6^ Taï Chimpanzee Project, Centre Swiss for Scientific Research Abidjan 1303 Ivory Coast; ^7^ UMI SOURCE, Université Paris‐Saclay, UVSQ, IRD Guyancourt 78280 France; ^8^ INRAE, VetAgro Sup, UMR Herbivores, Université Clermont Auvergne Clermont‐Ferrand 63000 France; ^9^ Institut Polytechnique UniLaSalle, IDEALISS, Transformations et Agro‐Ressources ULR 7519, Université d'Artois, Collège vétérinaire Mont‐Saint‐Aignan 76130 France; ^10^ Department of Evolutionary Anthropology University of Zurich Zurich 8057 Switzerland; ^11^ Center for the Interdisciplinary Study of Language Evolution University of Zurich Zurich 8050 Switzerland; ^12^ Wild Minds Lab, School of Psychology & Neuroscience University of St Andrews St Andrews KY16 9JP UK; ^13^ Department of Psychology Hunter College, CUNY New York 10065 New York USA

**Keywords:** comparative research, facial signal, gesture, multimodality, pragmatics, vocalisation

## Abstract

The concept of flexibility in communication is central to reconstructing the evolutionary history of language, and grappling with “contextual flexibility” in particular is pivotal to address implications for pragmatics‐first accounts of language evolution. Despite significant advances in the field, research is hindered by definitional inconsistencies and methodological gaps across modalities. We build on recent frameworks to propose further, detailed methods for studying contextual and functional variability, incorporating modality‐agnostic and standardised terminology to facilitate cross‐species comparisons. Our approach includes a detailed classification of communicative contexts and outcomes, offering practical methods to disentangle context from function and meaning. By integrating insights across behavioural ecology and comparative psychology, we aim to enhance the comparability of findings and provide a robust foundation for exploring the evolutionary trajectory of communicative flexibility and pragmatics.

## I. INTRODUCTION

Behavioural flexibility broadly refers to an individual's ability to adjust their behaviour, and reflects the degree to which behaviour is produced voluntarily rather than in response to a stimulus (Coppens, de Boer & Koolhaas, 2010). As humans, we are extraordinarily flexible in our communicative behaviour, producing diverse signals in diverse modalities and diverse combinations across diverse interactional scenarios towards diverse meanings (Kendon, 2004; Levinson & Holler, 2014; Scott‐Phillips, 2014). Given its relevance for human language, comparative researchers have been justifiably interested in the extent of flexibility in other species' communication (e.g. Call & Tomasello, 2007; Arbib, Liebal & Pika, 2008; Seyfarth & Cheney, 2018). Research on non‐human primates, in particular, has found, as in humans, that often there is no one‐to‐one correspondence between signal and function (Clark *et al*., 2020; Graham, Furuichi & Byrne, 2020; Rebout *et al*., 2020; Aychet, Blois‐Heulin & Lemasson, 2021a; Fröhlich *et al*., 2021b; Taylor *et al*., 2023). Defying the traditional code‐based model of animal communication (Scott‐Phillips, 2014; Cartmill, 2023), primate communication seems to be highly context sensitive (e.g. Wheeler & Fischer, 2012; Seyfarth & Cheney, 2018), lending support to pragmatics‐first theories of language evolution (Bar‐On & Moore, 2017; Moore, 2017; Scott‐Phillips, 2017).

If this increased research attention has been critical to advance the field, it has come at the expense of definitional and terminological clarity. The literature is replete with confusion. In the past, flexible use has been inferred from using the same signal across *contexts* (i.e. the setting or circumstances of a communicative act) and across (purported) *functions* (i.e. the purpose or information conveyed by a communicative act). Because initial systematic studies of gesture use operationalised flexibility as variation in signal use across concurrent general behaviour (“means–end dissociation”), primate gestural communication has been widely touted as a far more “flexible” communication system than primate vocal communication (e.g. Corballis, 2002; Arbib *et al*., 2008; Tomasello, 2008). However, more recent studies examining variability in vocal and multimodal communication challenge this idea (e.g. Slocombe, Waller & Liebal, 2011; Liebal *et al*., 2013; Levinson & Holler, 2014; e.g. Cheney & Seyfarth, 2018). We therefore need integrative definitions of signal “context” and “meaning” that can be applied across modalities. To elucidate the origins of context sensitivity in communication, we must clarify and operationalise key concepts, as has been achieved in recent work on terminology (Amphaeris *et al*., 2023; Berthet *et al*., 2023). Alongside these definitional operationalisations, we must scrutinise current approaches to studying communicative flexibility with the aim of developing methodological frameworks for truly comparative research across species, systems, and researchers.

The purpose of this paper is to do this, in three ways. First, we revisit the use and operationalisation of “flexibility”, “context”, and “meaning” within comparative research on primate communication. We highlight discrepancies among research domains (facial, gestural, vocal), which hamper comparability and integration of evidence for flexible signal use in multimodal studies. Second, building on recent theoretical work (Watson *et al*., 2022; Amphaeris *et al*., 2023; Berthet *et al*., 2023), we offer a new classification of contexts and meanings, proposing a framework with which to study contextual flexibility. Third, we discuss the implications of a rigorous investigation of flexibility in animal communication for language evolution and outline how this new framework can inform research on non‐human pragmatics.

## II. FLEXIBILITY *VERSUS* SPECIFICITY IN COMPARATIVE COMMUNICATION RESEARCH

### (1). Defining and assessing communicative flexibility

We first want to caution against discrepancies in terminology between behavioural ecology and comparative psychology on what is considered “flexible” and “fixed” (e.g. functionally specific) communicative behaviour. Behavioural ecologists characterise flexibility as *within‐individual* modification or adaptation of behaviour *in response to immediate environmental or social conditions* (Coppens *et al*., 2010), and thus a reversible form of behavioural plasticity (Gabriel *et al*., 2005; Dingemanse & Wolf, 2013; van Schaik, 2013). Comparative psychologists, on the other hand, typically characterise flexibility in communication as “means–end dissociation” – the use of one signal for several social goals and *vice versa*, usually assessed at the level of community or species (Tomasello *et al*., 1994).

These conceptualisations are at odds, as on the former behavioural ecology account not all behavioural variation within individuals qualifies as flexibility; other factors can drive variation in individual behaviour over repeated instances of interaction. For instance, within‐individual variation can also arise due to low individual predictability, that is, the degree of residual within‐individual behavioural variability after controlling for individual variation in average behaviour and individual plasticity (Biro & Adriaenssens, 2013; Westneat, Wright & Dingemanse, 2015). Individuals may vary consistently in their predictability, especially younger individuals who are in the exploratory phase of their communicative development (Boeckx, 2023), and who are also considered more behaviourally flexible than adults, having not yet arrived at a stable set of communicative “tools” (Fröhlich & Hobaiter, 2018). We argue that comparative psychologists (such as many of this papers' authors) should move to align our approach with behavioural ecology, in considering individual flexibility as adjustments *in response to* the communicative conditions of the signaller–recipient dyad.

Methodological approaches addressing flexibility in animal communication vary across modalities. While researchers studying gestural and facial signals have generally conducted observational studies of behaviour surrounding communication events to infer intentional signal use (e.g. Pika, Liebal & Tomasello, 2003; Liebal, Pika & Tomasello, 2006; Pollick & de Waal, 2007; Waller, Caeiro & Davila‐Ross, 2015; Aychet *et al*., 2021a, 2021b), work on vocalisations has predominantly deployed playback experiments to test hypotheses on the representation and functional reference of signals (Seyfarth, Cheney & Marler, 1980; Arnold & Zuberbühler, 2008; Wegdell, Hammerschmidt & Fischer, 2019). In both paradigms, early definitions of signal function focused on context – the circumstances that immediately surround a communicative act (although it is often unclear which temporal window is considered for these “immediate circumstances”, see Table 1).

**Table 1:** Definition of key terms used in comparative communication research, illustrated with three examples: a contact call, a scream, and an alarm call.

Behavioural context (e.g. Graham *et al*., 2020)	Activity of the signaller or group within a defined time window surrounding the signal occurrence, e.g. “travelling” (contact call), “receiving aggression” (scream) or “attending to leopard” (alarm call).
Environmental context (e.g. Wheeler & Fischer, 2012)	Biotic and abiotic environment surrounding but independent of the behavioural context, used by recipients to access information that enriches but is not actually conveyed by the signal type, e.g. “water crossing” (contact call), “supporter nearby” (scream) or “leopard proximity” (alarm call).
Function (e.g. Schamberg *et al*., 2018)	Known or hypothesised general benefit of a signal, e.g. “increased group cohesion” (contact call), “conflict avoidance/social bonding” (scream), “predation avoidance” (alarm call).
Meaning	Inferred goal on the occasion of signal use, and thus change in the recipient's behaviour that the signaller intends, e.g. “travel with me” (contact call), “support me” (scream), “move away from predator” (alarm call).
Outcome (e.g. Hobaiter & Byrne, 2014)	The behaviour of the potential recipient(s) that follows the signal, e.g. “travels together” (contact call), “gives social support” (scream) or “climbs up a tree” (alarm call). Note that a behavioural *change* is not always expected (e.g. for signals of benign intent or submission).

Extensive efforts to describe great ape gesture repertoires thoroughly within the scope of observational studies resulted in gestures being characterised as extraordinarily flexible (Call & Tomasello, 2007; Arbib *et al*., 2008), with substantial degrees of means–end dissociation. In primate vocalisation studies, the quest for functionally referential calls has often focused on “context‐specific” signals (i.e. signals observed only in single scenarios), leaving observations of contextually variable calls (i.e. signals observed in multiple scenarios) largely unexamined or unpublished (Wheeler & Fischer, 2012; but see Clay, Archbold & Zuberbühler, 2015; Rebout *et al*., 2020; Taylor *et al*., 2022). Similarly, facial signals are often considered to be tied to specific contexts and functions (but see Duboscq *et al*., 2013; Clark *et al*., 2020). But as interest in communicative “flexibility” has increased, several primate species are now known to use certain facial and vocal signals in more than one interactional scenario or “context” (Flack & de Waal, 2007; Duboscq *et al*., 2013; Clay *et al*., 2015; Clark *et al*., 2020; Kim *et al*., 2022; Taylor *et al*., 2022, 2023).

### (2). Operationalising flexibility for signal meanings

A central challenge in the study of non‐human animal communication, which seems to be at the core of this conflation problem, has been determining what signals “mean” and/or whether they can be said to have “meaning” in the first place (Scott‐Phillips, 2015; Amphaeris *et al*., 2023). Nonetheless, we now see broad agreement that signal meanings can be determined in communication systems where signals are goal‐directed, with the signaller aiming to change the recipient's behaviour (e.g. Moore, 2015; Byrne *et al*., 2017; Seyfarth & Cheney, 2017; Amphaeris *et al*., 2023), so‐called “first‐order intentionality” (Dennett, 1983). Studies focusing more on audience checking, sensitivity to visual attention, and persistence provide evidence that using signals in non‐human primates can be intentional and voluntary (e.g. Bard, 1992; Liebal *et al*., 2004; Leavens, Russell & Hopkins, 2005).

To ascertain the goal‐directedness of signals, several primate researchers (e.g. Hobaiter & Byrne, 2014; Graham *et al*., 2018) have focused on *Apparently Satisfactory Outcomes* (ASOs): changes in recipient behaviour after which signalling stops, thus reflecting plausible social goals. The presence of an intended outcome implies that the link between the signal and behavioural outcome is potentially uncoupled (i.e. not 1:1) and that the signal is primarily linked to the goal. The *general effect* (“function”, Table 1) becomes the *intended effect* (“meaning”, Table 1) when the signaller represents the goal state, while the exact meaning depends on signal type. For example, while the sexual swelling of an oestrous chimpanzee female has a clear function, we also see a meaningful signal when this female moves to expose this swelling actively to a visually attentive male to solicit copulation (i.e. through a “present” gesture). Or, while a scream vocalisation may have the general effect of recruiting allies, it would have an intended effect and thus meaning if it is directed at specific bystanders (e.g. bonding partners or kin) with the intention of recruitment. In sum, while *function* corresponds to the impact of the signal that represents, on average, a benefit for the signaller and recipient (Table 1), *meaning* can emerge as a change in the recipient's behaviour that was intended by the signaller (Table 1) (Byrne *et al*., 2017).

To gain insight into context sensitivity in signal meaning, comparative researchers have increasingly studied recipient behaviour and interaction outcomes (Wiper & Semple, 2007; Cartmill & Byrne, 2010; Waller & Cherry, 2012; Schel *et al*., 2013b; Hobaiter & Byrne, 2014; Graham *et al*., 2018; Clark *et al*., 2020; Fröhlich *et al*., 2021a). This approach has revealed variation in the degree of specificity of ape gestures, with some gestures being functionally specific and others functionally variable (Hobaiter & Byrne, 2014), the latter being disambiguated mainly using cues provided by the signaller's prior activity (Graham *et al*., 2020). While the ASO methodology is useful, not all signals result in an immediate change in a specific recipient's behaviour, for example contact calls. Likewise, food grunts in chimpanzees *Pan troglodytes* may be addressed to particular individuals or to a large audience (Schel *et al*., 2013a) without any clear way for researchers to know the target recipients. In fact, it is possible that grunts in these different social settings may be subtly different – a possibility that has also been raised for certain facial signals (Clark *et al*., 2020). Fortunately, recent advances in data technologies, for example tools like DeepLabCut for automated pose estimation (Wiltshire *et al*., 2023) are allowing researchers to investigate more subtle differences in signal variation. Yet while we are making headway in assessing variation in signal types and meanings, we have failed sufficiently to tackle the big muddy concept of context.

### (3). Operationalising flexibility for communicative contexts

Not only are there biases in research questions and approaches for specific modalities, the concept of “context” itself lies at the core of the discrepancy between facial, gestural, and vocal signalling research. This state of affairs is obviously problematic, particularly when the goal is to understand what information is relevant for interpreting signal meanings (Zuberbühler & Neumann, 2017). If context is critical to approximating “meaning” in facial, gestural, and vocal communication, we must make a clear distinction between signal types that are produced in a range of contexts (contextual variability) and those that can be created to fulfil several communicative functions at different times (functional variability). For example, contact calls (e.g. the hoo of chimpanzees, the coo of macaques) may be contextually variable, as they are often produced in multiple contexts (Arlet *et al*., 2015; Clay *et al*., 2015; Rebout *et al*., 2020), but their function is nonetheless specific: maintaining or re‐establishing group cohesion.

Researchers commonly use context to infer what circumstances elicit a signal (e.g. Crockford & Boesch, 2003), but there are (at least) two different primary uses of the term (*cf*. Zimmermann, Lorenz & Oppermann, 2007). In the first usage, context is specified by enumerating elements related to additional information sources, including all available information *not* included in the signal (Zuberbühler & Neumann, 2017). These contextual elements are mainly related to the activity or social interaction in which a signaller is engaged (behavioural context, Table 1) and often include major behavioural categories like feeding, grooming, playing, resting, and sex (Call & Tomasello, 2007; Liebal *et al*., 2013; Graham *et al*., 2020). In the second usage, context is defined by specifying the information that surrounds the referent (i.e. environmental context, Table 1) but that is not directly referred to (e.g. when someone says “door!”, the state of the door informs whether you are being instructed to close, open, or simply look at it). For example, a recent review on vocal learning defined usage learning as the learning of the environmental context (e.g. a specific set of predators) in which to employ a signal (Vernes *et al*., 2021). Further confusion is caused by using the terms “context”, “environment”, and “situation” synonymously, thereby creating self‐referencing loops (Zimmermann *et al*., 2007). Thus, the first way of specifying context (“by example”) aims to address a full set of features that are “contextual”, while the second approach (“by synonyms”) does not, which can only lead to confusion (Zimmermann *et al*., 2007). For example, definitions “by example” typically highlight contextual information related to the signaller's activity, such as the social interaction the signaller is engaged in. By contrast, definitions “by synonyms” generally focus on *one* specific situational aspect that may be relevant to the signaller, such as the presence of a predator.

A few studies have characterised behavioural contexts by positive, neutral, or negative affective or valence states elicited in interacting subjects (Rebout *et al*., 2020; Aychet *et al*., 2022; Taylor *et al*., 2023). These distinctions have been particularly relevant for facial communication studies, which have historically assumed strong links between signals and emotions (Darwin, 1872/2013), with facial signals treated as a consequence of social and environmental context rather than a driver of interactions. While these assumptions likely oversimplify facial communication (Van Hooff, 1967; Barrett *et al*., 2019), the operationalisation of context for facial communication research may yet inform how we operationalise context across modalities.

In the big picture, definitions of context diverge both between and within facial, gestural, and vocal communication research domains. This lack of comparability limits the conclusions that we can derive from our studies, especially where “context” is used as a proxy for signal function (Clay *et al*., 2015; Aychet *et al*., 2021a). In one interpretation, a signal may be contextually variable because it is produced across several high‐arousal situations like predator threat or aggression. In another interpretation, the same signal is contextually specific because it is only produced by individuals with a negative valence state. In the strict sense, however, a signal's function refers to its evolved effect (Maynard Smith & Harper, 2003; Laidre & Johnstone, 2013) and thus should not be inferred from the behaviour or environment surrounding its production alone. Explicitly disentangling context from function or meaning should be a critical step to avoid this confusion.

## III. STANDARDISING THE STUDY OF CONTEXT IN COMPARATIVE COMMUNICATION RESEARCH

So far, we have introduced the concept of flexibility and proposed definitions for terms where we might examine variability that is relevant to semantic and pragmatic communication in other species. As such, Table 1 spells out the key distinctions of the concepts needed for the study of pragmatics in non‐human species. Context, however, is currently a much cloudier concept lacking in standardised assessment. In the previous section, we overviewed ways in which context has so far been operationalised. In this section, we present a more comprehensive and practical categorisation of context. We argue that this should include all information available to signaller and recipient that is relevant to communication, such as: signaller/recipient behaviour before/after the signalling event; the shared interaction history with the recipient and their social/kin/dominance relationship; and age/sex combinations (Fröhlich, Wittig & Pika, 2016; Graham *et al*., 2020; see Table 2).

**Table 2:** Classification of our proposed types and subtypes of context which may alter signal production and/or meaning in non‐human species.

Type of context	Sub‐type	Definition	Study examples
Interactional	Signaller behaviour	The behaviour of the signaller immediately (within specified time window or behavioural cues) before and whilst producing the signal	Graham *et al*. (2020) (bonobos *Pan paniscus*): signaller's behaviour immediately before and during signal production modulates interaction outcomes
	Recipient behaviour	The behaviour of the recipient immediately (within specified time window or behavioural cues) before the signal is produced	Flack & de Waal (2007) (pigtailed macaques *Macaca nemestrina*): prior recipient behaviour modulates signal function
	Recent shared interaction history (within the same interaction)	Within a set period, the nature and rate of social interactions between signaller and recipient	Cartmill & Byne (2007) (orangutans *Pongo abelii/pygmaeus*): signaller modulates signal depending on recipient's response to previous signal
	Recipient(s) attention	Whether the intended recipient and/or other individuals are attending to the signaller	Waller *et al*. (2015) (orangutans): presence of play‐face affected by recipient's visual attention state
	General audience (no clear single recipient)	Presence and abundance of individuals in party/subgroup	Ota *et al*. (2018) (blue‐capped cordons‐bleus *Uraeginthus cyanocephalus*): multimodal courtship display depending on the presence of an audience
Social	Age difference	Precise age gap (in months or years) between signaller and recipient (can be alternatively collapsed to age group difference)	Fröhlich *et al*. (2016) (chimpanzees), Fröhlich *et al*. (2021b) (orangutans): signal modality varies according to age difference (e.g. peers)
	Sex combination	Sex of signaller and recipient	Graham *et al*. (2020) (bonobos): sex combination between signaller and recipient affects the outcome of specific gestures in bonobos
	Relative dominance	Relative rank position or power rating between signaller and recipient	Clark *et al*. (2022) (macaques): relative rank affects use of bared‐teeth variant in Sulawesi crested macaques *Macaca nigra*
	Kin relationship	Relatedness of signaller and recipient, potentially standardised for whole group	Fröhlich *et al*. (2016) (chimpanzees), Fröhlich *et al*. (2021b) (orangutans): kinship affects gesture modality
	Social relationship	Affiliation index	Kern & Radford (2016) (dwarf mongooses *Helogale parvula*): social bond strength affects responses to recruitment calls
Internal	Emotional or valence state	Valence state of signaller (e.g. positive, neutral, negative)	Clay *et al*. (2015) (bonobos): valence state predicts acoustic structure of “peep” call
	Signaller or recipient physiological state	Stage of menstrual cycle (hormonal levels, sexual swelling, etc.)	Roberts & Roberts (2015) (chimpanzees): recipient's menstrual state affects signal modality; Demuru *et al*. (2020) (bonobos): signaller's sexual swelling affects their postural behaviours
Environmental	Predator or threat presence	Perceived threat by aerial or terrestrial predators, or adult males in the group (potential infanticide risk)	Price *et al*. (2015) (vervet monkeys *Chlorocebus pygerythrus*): predator type modulates use and structure of vocalisations
	Habitat type	Type of habitat where the signaller and recipient are currently (e.g. background contrast)	Hulse *et al*. (2020) (darter fish *Etheostoma* spp.): background contrast affects sexual signal pattern
	Vegetation density	Visibility distance as an estimate of vegetation density around signaller and recipient	Nicholls & Goldizen (2006) (bowerbirds *Ptilonorhynchus violaceus*): habitat type and vegetation density affect vocal signal
	Strata/position	Vertical location (and position) of signaller and recipient	Lameira & Call (2018) (orangutans): height modulates probability of alarm call
	Weather	Current weather during signal	Lengagne & Slater (2002) (tawny owls *Strix aluco*): rain leads to reduced call rates
	Anthropogenic conditions	Nearby human activities modifying sound environment, like traffic and industry noise	Duarte *et al*. (2018) (titi monkeys *Callicebus nigrifrons*): mining noise affects structure and frequency of “loud calls”
	Individuals other than the group	Interaction with or observation of out‐group conspecifics/allospecifics	Persson *et al*. (2018) (chimpanzees): mutual imitation of gestures and facial signals between zoo visitors and chimpanzees

Table 2 outlines (sub)types of contexts that have been shown to be relevant to non‐human communication and includes one or two studied examples each, and thus describes in more detail different ways in which two key notions in comparative communication research – the environmental and behavioural contexts introduced in Table 1 – can be elaborated. We organise subtypes into four overarching context categories: interactional, social, internal, and environmental. Interactional context includes the behaviour of the signaller and recipient prior to signalling, their shared interaction history, the recipient's attention, and the presence/composition of the audience. Additionally, one might consider the behaviour of the audience, but we found no evidence of this effect in the literature, so we did not include it in the table. Social context includes age, sex, rank, kin, and social relationships between signaller and recipient. Internal context includes the physiological and affective states of the signaller and recipient. Environmental context includes variables beyond the signaller, recipient, and audience, such as the presence of predators, habitat type, vegetation density, strata, weather, human activity, and out‐group interactions (e.g. zoo visitors). Of these types of context, internal and environmental context have been studied the least in terms of communication, and so these categories are likely more exploratory than interactional and social contexts. Note that not all of these represent shared context in the sense of information being available to both signaller and recipient. While social and interactional contexts are likely to be largely shared, relevant environmental and internal information may often be accessible to only one member of the dyad.

While our framework aims to standardise definitions and categories, obviously researchers should tailor this to their needs and should be transparent and explicit when doing so. For example, researchers must consider markers to define the interactional context, which is either temporal (e.g. 3 s: Rebout *et al*., 2020) or behavioural (e.g. when an individual leaves her resting position before engaging in grooming a conspecific, the interactional context is labelled “rest” rather than “travel”, since co‐locomotion served merely to approach this conspecific: Graham *et al*., 2020). If a time window is considered more suitable than behavioural cues for associating some observed behaviours to the signal events, it must be adapted to the species, the communicative modality, and the research question. There may also be other types of context that are relevant to certain species, and we encourage researchers to consider what contextual information their study subjects may be drawing on in their communication.

The critical steps, therefore, would be to: (*i*) identify the types of information that enrich context, which may differ across species or modalities; (*ii*) verify that these contextual cues (e.g. bystander presence) can be reliably assessed; and (*iii*) include them as predictive variables for the production of different signals in a data set. Such an approach could test whether certain gestures or calls are used specifically or variably in certain environmental situations (e.g. where visibility is poor) or in more or less emotionally charged social interactions (e.g. screams that are modified in relation to the audience: Slocombe & Zuberbühler, 2007). Quasi‐experimental observational research paradigms, such as analysing individual variation in different environmental, social or interactional settings, will help to elucidate the sources of information that animals use to select appropriate signal types/modalities and to disambiguate signal meanings. In turn, this may help to resolve questions about signal function, by determining whether certain signals are functionally similar but used in different environmental or social interactions. Importantly, this may suggest alternative (non‐functional) explanations for why some signals are sometimes preferred over others.

Why might such a fine‐grained approach to classifying context be important for comparative researchers? First, a closer look at context(s) may lead us to conclude that many signals currently thought to have multiple functions (or outcomes) may actually have a single function, and *vice versa*. For example, some of the chimpanzee gestures described by Hobaiter & Byrne (2014) are listed as having multiple outcomes (e.g. move away, move towards) when in fact they might have a single function: move (Moore, 2014). At the same time “move away” and “move towards” seem to be outcomes of highly contrasting interactive scenarios (i.e. involving agonistic *versus* affiliative social goals). This issue is particularly relevant for directive tactile signals, such as the gestures *touch*, *push*, and *pull*. We hope that the conceptual clarifications herein will help to resolve such questions and provide new insights into whether signals have fixed semantic properties. A careful distinction between context, function and meaning is critical to determine the extent to which animal communication can be modelled by a simple code or to what extent they may be making pragmatic inferences (Bohn *et al*., 2022; Cartmill, 2023).

## IV. INSIGHTS FOR FUTURE RESEARCH ON FLEXIBILITY IN EVOLUTIONARY PRAGMATICS

In primates and other long‐lived social species where the same individuals interact repeatedly, communication constitutes a “rich system of pragmatic inference in which the meaning of a communicative event depends on perception, memory and social knowledge” (Seyfarth & Cheney, 2017, p. 339). Our review puts forward three major insights for future research on evolutionary pragmatics and language evolution.

(1). Flexibility should not be reported as a presence/absence feature of a communication system and should not be conflated with variability; rather, we should quantify variability in more detail across communicative parameters (Tables 1 and 2). Rather than trying to estimate flexibility by assessing how many signal types are produced in specific contexts, we should instead study the variable relationships between signals and specific functions, meanings, outcomes, and contexts on an *individual* level. Understanding the conditions under which different types of flexibility evolve and emerge within communication will require this kind of broad, standardised, comparative approach. As such, our proposal dovetails with a recent framework for “optionality”, which describes the capacity to associate alternative functions with a signal or alternative signals with a function across “signal production, signal adjustment, signal usage, signal combinatoriality and signal perception” (Watson *et al*., 2022).
(2). Signal context and signal function (or meaning or outcome) should not be conflated; rather, they should be studied separately. This is improving across the field, but researchers must commit to clarifying further whether they are reporting context or function (or both). We make proposals for how to describe context, function, meaning, and outcome (Tables 1 and 2). By identifying the intended change in the recipient's behaviour (Graham *et al*., 2018; Amphaeris *et al*., 2023), we may be able to make predictions about the presence (or absence) of particular social outcomes (see online Supporting information, Table S1, for a standardised list of signal functions and outcomes in chimpanzees) irrespective of signal type (e.g. contact signals, imperative requests, functionally referential signals) and modality (gestural, facial, vocal), strengthening research comparability.
(3). The comparative study of communicative flexibility requires both a *multi‐level* and *multimodal* approach. By multi‐level, we mean at the levels of individual, species, and population (Fröhlich *et al*., 2022); and by multimodal, we mean across all communication systems within the study organism, for example facial, gestural, and vocal communication. As flexible signal use is an individual capacity, claims of species‐level or population‐level (e.g. “dialect”) differences in pragmatic inference capacity should naturally consider variation in signal use at the individual level. Future research that uses our standardised operationalisation of signal contexts and functions can assess better the extent to which multiple and multimodal signals are contextually variable (i.e. elicited by different social or environmental situations: Aychet *et al*., 2021a) and functionally variable [i.e. leading to different social outcomes depending on the context and structure of signal associations (Genty *et al*., 2014; Fröhlich *et al*., 2021b)].

These three concerns are exemplified in recent work by Bohn *et al*. (2022) who used the Rational Speech Act framework (Frank & Goodman, 2012) to model chimpanzee communicative interactions as a social inference process, in which receivers rationally integrate multiple information sources (gestures, facial signals, social context). Since the initial model refrained from specifying the cognitive processes underlying each information source (e.g. intentions), Franke, Bohn & Fröhlich (2024) built on this work by using formally specified probabilistic models of production and interpretation behaviour. This allowed them to investigate contextual flexibility by comparing signal meanings in great apes living in different communicative settings (wild *versus* captive setting; mother–offspring dyads *versus* other relationships). Further work is needed to integrate better a wider range of affiliative, rank and kin relationships and thereby consider the interactional and social context. While evidence for the role of environmental context beyond predators or perceived threat is still scarce, a holistic perspective on the notion of context will help to assess more rigorously the setting within which signals are produced.

Through developing this framework, we have identified important avenues for further research. First, given that signals can be classified by form (structural communication units) or outcome (functional communication units), it is still unclear how we can address the comparability and transparency of communicative repertoires across studies. In other words, how do we distinguish signals from morphological variants with the same function? Second, it is still unclear whether co‐occurring signals produced in another modality by the same signaller comprise a context for a given signal, or whether context should always be external to the communicative act. Third, a central goal should be to mitigate observer bias, considering that depending on the amount of experience with observing interactions of the same individuals within a particular social group, researchers may naturally be biased about what they think is the relevant interactional or social context. As this limits our opportunities for fair inter‐rater reliability tests, we need to find ways to consider differential observer knowledge on previous interactions systematically.

Finally, we believe it is timely to integrate emotions, affect, and valence into the discussion on context and meaning. Human facial expressions (e.g. “fear displays”) may be associated with an affective experience (Ekman *et al*., 1992; Shariff & Tracy, 2011) but can also serve as strategic social tools (Crivelli & Fridlund, 2019). Similarly, animal signals can be both emotional *and* voluntarily produced (Marler, Evans & Hauser, 1992; Dezecache, Mercier & Scott‐Phillips, 2013; Heesen *et al*., 2022), highlighting the need to acknowledge both extraneous context and intrinsic factors like emotional states. Technological advancements, such as thermal imaging (Brügger, Willems & Burkart, 2021; de Vevey *et al*., 2022), will likely help to take these better into account as contextual elements (Table 2).

## V. CONCLUSIONS

(1). Operational definitions for context, function, outcome, and meaning – and their subtypes – are often overlooked as tools for studying the origins of human language, including pragmatics and semantics. While researchers put great effort into standardising and making transparent experimental and observational data‐collection protocols, it is equally important to standardise and make the definitions of terms transparent.
(2). The comparative approach may be a powerful tool to unravel the origins of humans' exceptional communicative plasticity (Liebal *et al*., 2013; Seyfarth & Cheney, 2018; Taylor *et al*., 2022), but progress in this field will depend on our efforts to study non‐human communication more holistically, recognising that comparative studies of contextual or functional variability will always require multiple levels of analysis. Such a research programme will rely on adopting a modality‐agnostic and comparable terminology.
(3). A standardised approach should be applied to a greater diversity of species within and beyond the primate order, in a range of captive to wild settings, and using experimental and observational techniques. Here, we have aimed to provide basic definitions for context, function, meaning, and outcome and a framework with which to study these. We hope that our research community will make use of this framework and that through ongoing large‐scale collaboration it will help us to understand the evolutionary trajectory of communicative flexibility.

## Supporting information


**Table S1.** Suggested signal functions and their observed outcomes in chimpanzee communication studies [signal definitions: Parr *et al*. (2007), Hobaiter & Byrne (2011), Crockford (2019)].
