# Supplementary material for: Assessing flexibility in meaning and context in non‐human communication
Source: Biol Rev Camb Philos Soc. 2025 Jul 23;100(6):2471–81. doi: 10.1111/brv.70054 (PMC12586293; doi:10.1111/brv.70054)
Supplement: Supplementary file 1 — Table S1. Suggested signal functions and their observed outcomes in chimpanzee communication studies [signal definitions: Parr et al. (2007), Hobaiter & Byrne (2011), Crockford (2019)]. [file BRV-100-2471-s001.docx]

**Supporting information**

**Assessing flexibility in meaning and context in non-human communication**

Marlen Fröhlich, Juliette Aychet, Peter R. Clark, Catherine Crockford, Guillaume Dezecache, Nancy Rebout, Carel P. van Schaik, Kirsty E. Graham

**Table S1.** Suggested signal functions and their observed outcomes in chimpanzee communication studies [signal definitions: Parr *et al.* (2007), Hobaiter & Byrne (2011), Crockford (2019)].

| **Function** | **Outcome in recipient** | **Example (chimpanzees)** |
| --- | --- | --- |
| Confirm dominance | Retreats from signaller | Branch-shaking display (gesture) |
| Confirm subordinate status | Tolerate signaller’s presence | Pant-grunt (vocalisation), bared-teeth display (facial signal) |
| Continue grooming session | Continue getting groomed | Lip-smacks during grooming (unvoiced vocalisation) |
| Continue play session | Continues playing with signaller | Play face (facial signal), laugh (vocalisation) |
| Elicit consolation | Reassure signaller | Whimper (vocalisation) |
| Engage in social play | Starts play with signaller | Slap object (gesture) |
| Maintain bond | Calms down after aggression | Embrace (gesture) |
| Maintain cohesion | Stays close to signaller | Travel hoo (vocalisation) |
| Mate with conspecific | Copulates with signaller | Beckon (gesture) |
| Prolong affiliation | (Re-)starts to groom signaller | Loud scratch (gesture) |
| Recruit of others to food | Approaches signaller | Deep grunt (vocalisation) |
| Recruit supporters | Supports signaller (during or after being attacked) | Victim scream (vocalisation) |
| Repel others | Stop present action | Roar pant-hoot (vocalisation) |
| Solicit object of desire (e.g. food item) | Transfers object | Reach (gestures), whimper (vocalisation) |
| Threatening competitors | Flees signaller, gives away resource | Branch-shaking (gesture), threat face (facial signal), roar pant-hoot (vocalisation) |
| Warning of threat | Attends to object, halts approach, or moves to safe place (e.g. shelter, canopy) | Alert hoo and other alarm calls (vocalisation) |

**References**

Crockford, C. (2019). Why does the chimpanzee vocal repertoire remain poorly understood and what can be done about it? In *The Chimpanzees of the Taï Forest: 40 Years of Research* (ed. C. Boesch and R. Wittig), pp. 394–409. Cambridge University Press, Cambridge.

Hobaiter, C. & Byrne, R. W. (2011). The gestural repertoire of the wild chimpanzee. *Animal Cognition* **14**, 747–767.

Parr, L. A., Waller, B. M., Vick, S. J. & Bard, K. A. (2007). Classifying chimpanzee facial expressions using muscle action. *Emotion* **7**, 172.
